# Absence of α-synuclein affects dopamine metabolism and synaptic markers in the striatum of aging mice^☆^

**DOI:** 10.1016/j.neurobiolaging.2008.11.001

**Published:** 2010-05

**Authors:** Abdelmojib Al-Wandi, Natalia Ninkina, Steven Millership, Sally J.M. Williamson, Paul A. Jones, Vladimir L. Buchman

**Affiliations:** a School of Biosciences, Cardiff University, Museum Avenue, Cardiff CF10 3US, United Kingdom; b Institute of Physiologically Active Compounds, Russian Academy of Sciences, 1 Severnyj Proezd, Chernogolovka, Russian Federation; c Astellas CNS Research Institute, University of Edinburgh, The Chancellor’s Building, 49 Little France Crescent, Edinburgh EH16 4SB, United Kingdom

**Keywords:** Synuclein, Null mutant, Synucleinopathy, Parkinson’s disease, Ageing, Dopaminergic neurons, Substantia nigra, Neuroprotection, Synaptoprotection

## Abstract

Despite numerous evidences for neurotoxicity of overexpressed α-synuclein, a protective function was suggested for endogenous α-synuclein and other members of the synuclein family. This protective role is most important for and evident in presynaptic terminals, where synucleins are normally accumulated. However, mice lacking synucleins display no adverse phenotype. In particular, no significant changes in striatal dopamine metabolism and only subtle deficit of dopaminergic neurons in the substantia nigra were found in juvenile or adult mice. To assess whether aging and synuclein deficiency may have additive detrimental effect on the nigrostriatal system, we studied dopaminergic neurons of the substantia nigra and their striatal synapses in 24–26-month-old α-synuclein and γ-synuclein null mutant mice. Significant ∼36% reduction of the striatal dopamine was found in aging α-synuclein, but not γ-synuclein null mutant mice when compared to age-matching wild type mice. This was accompanied by the reduction of TH-positive fibers in the striatum and decrease of striatal levels of TH and DAT. However, no progressive loss of TH-positive neurons was revealed in the substantia nigra of synuclein-deficient aging animals. Our results are consistent with a hypothesis that α-synuclein is important for normal function and integrity of synapses, and suggest that in the aging nervous system dysfunction of this protein could become a predisposition factor for the development of nigrostriatal pathology.

## 1 Introduction

The decade of intensive studies that followed the original demonstration of direct links between α-synuclein and Parkinson’s disease (Polymeropoulos et al., 1997; Spillantini et al., 1997) revealed structural, functional and metabolic properties of this protein that might be responsible for its role in the development of Parkinson’s and other neurodegenerative disorders, now known as synucleinopaties. Nevertheless, the question about exact mechanism of α-synuclein involvement in neurodegeneration is still wide open. Histopathological observations together with high propensity of α-synuclein to aggregate, which could be further increased by certain causative mutations, led to an intuitive hypothesis that aggregation into large insoluble filaments, which become major constituents of Lewy bodies and other inclusions, is the primary cause of neurodegenerative changes (Trojanowski et al., 1998; Spillantini and Goedert, 2000; Galvin et al., 2001). However, further clinical and experimental studies questioned the importance of the final products of α-synuclein aggregation, fibrils or filaments, for the cytotoxic effect of mutated or overexpressed α-synuclein (Mori et al., 1998; Saha et al., 2000; Volles et al., 2001; Gosavi et al., 2002; Volles and Lansbury, 2002). It has been suggested that although enhanced aggregation is a prerequisite for α-synuclein cytotoxicity, the toxic effect should be attributed not to fibrils but to soluble intermediates of aggregation process, oligomers or protofibrils (reviewed in Dev et al., 2003; Volles and Lansbury, 2003). Moreover, a swing of aggregation kinetics in favour of the final steps of the process could reduce intracellular concentration of these toxic α-synuclein species by precipitating and trapping them within the insoluble deposits. Aggregation products could be cleared up to a certain extent, by intracellular systems (e.g. autophagosome and lysosome) that are different from systems metabolising soluble proteins (e.g. proteasome), which increases the ability of cells to combat the increased production of potentially toxic proteins like α-synuclein (Ciechanover and Brundin, 2003; Webb et al., 2003; Cuervo et al., 2004; Lee et al., 2004; Rideout et al., 2004). Although toxicity of aggregation intermediates is currently widely accepted as the major step in the development of α-synuclein-induced pathology, other mechanisms may also play an important role in this process. One significant consequence of aggregation or aberrant intracellular trafficking of α-synuclein is depletion of its pool at the intracellular sites where this protein is normally localised, primarily in the presynaptic terminals. The outcome of the resulting loss-of-function is not apparent because above all the function itself is still not defined. However, recent studies clearly demonstrated the importance of α-synuclein in protection from at least some types of synaptic dysfunction and consequent neurodegeneration (Chandra et al., 2005). This function of α-synuclein could be revealed only in a case when certain other protective mechanisms fail, suggesting an auxiliary role of this protein in neuroprotection. Studies of various tissue culture models also demonstrated that α-synuclein could act as a protective factor but only in particular conditions and types of cells (reviewed in Dev et al., 2003). Moreover, animals lacking functional α-synuclein gene as the result of either spontaneous deletion or targeted inactivation do not show any signs of degeneration in their nervous system (Abeliovich et al., 2000; Specht and Schoepfer, 2001; Cabin et al., 2002; Dauer et al., 2002; Schluter et al., 2003). A simple functional compensation by other members of the family, β-synuclein and γ-synuclein, could not explain this lack of pathological changes because neither double nor triple synuclein adult null-mutant mice develop adverse phenotype (Chandra et al., 2004; Robertson et al., 2004; Papachroni et al., 2005 and our unpublished observations). However, no studies of the nervous system of aging synuclein null mutant mice have been carried out so far. Aging is associated with functional decline of many protective systems within cells. In particular, age-related changes in the nigrostriatal system could predispose to the development of Parkinson’s disease (Phinney et al., 2006; Chu and Kordower, 2007; Collier et al., 2007). It is feasible that the absence of auxiliary protective factors like synucleins might exacerbate pathological changes related to the senescence of the nervous system. This is consistent with clinical data suggesting that aging is the major risk factor for developing idiopathic synucleinopathies.

We studied the nigrostriatal system of aging synuclein null mutant mice and demonstrated that the absence of α-synuclein but not γ-synuclein leads to the substantial decline of dopamine level and decrease of expression of certain synaptic markers in the striatum without reduction of the number of dopaminergic neurons in the substantia nigra pars compacta (SNpc).

## 2 Methods

### 2.1 Animals

Generation and maintenance of colonies of α-synuclein and γ-synuclein null mutant mice on pure C57Bl6J genetic background was described previously (Ninkina et al., 2003; Robertson et al., 2004). Experimental cohorts for this study were formed from litters produced by intercrossing heterozygous animals. After weaning and genotyping the null mutant and wild type male littermates were housed one per cage with free access to water and food until they reach age of 24–26 months. Animals (for simplicity refer in the manuscript as 2-year-old mice) were sacrificed by cervical dislocation, and tissues were collected and coded. Therefore, individuals that performed all further analyses were unaware of the sample genotype. Animal performance in an accelerating rotarod test was assessed as described previously (Robertson et al., 2004).

### 2.2 Neuronal cell counts

For quantification of neurons, brains of age-matching wild type, α-synuclein and γ-synuclein null mutant mice were collected, fixed, processed, embedded and stained simultaneously. 8-μm-thick sections were cut using a HM 310 microtome (Microm International) and mounted onto poly-l-lysine coated slides. The borders of the substantia nigra and ventral tegmental area (VTA) on histological sections were outlined using distribution atlas of tyrosine hydroxylase (TH)-positive cells (Hokfelt et al., 1984). The number of dopamine positive neurons in these two brain regions of 2-year-old animals was assessed by stereological counting of cells stained for TH on serial histological sections as described previously (Robertson et al., 2004). Briefly, the first section for counting was randomly chosen from the first 10 sections that included SNpc/VTA region. Starting from this section, every TH-positive cell with a clearly seen nucleus was counted on the every tenth section through the whole region. The Axiovision imaging program (Carl Zeiss Vision) was employed to measure diameters of 50 nuclei of dopaminergic neurons in the SNpc and 50 nuclei of dopaminergic neurons in the VTA of every mouse brain included in this study. The nuclei were chosen randomly and the distance measured as the horizontal length as they appeared on screen. A mean was calculated for each animal and used for Abercrombie’s correction (Abercrombie, 1946) to obtain an actual number of TH positive cells in the structure.

### 2.3 HPLC analysis of striatal dopamine and its metabolites

Striata of 2-year-old mice were dissected, immediately snap-frozen in liquid nitrogen and kept at −80 °C. Extraction and HPLC analysis of striatal dopamine and its metabolites was carried out as described in our previous publication (Robertson et al., 2004).

### 2.4 Density of immunostaining in striatum

Coronal brain sections at the level of striatum were immunostained with antibody against tyrosine hydroxylase (mouse monoclonal, clone TH-2, Sigma, diluted 1:5000). Signal detection was carried out according to previously described protocol (Benno et al., 1982). In each experiment, all sections were processed and immunostained simultaneously, and special care was taken to assure that the length of the development step was the same for each section. Images of stained sections were obtained using AlphaImager 2200 and densities of staining in the dorsal striatum were analyzed using AlphaEase 2200 Analysis Software (AlphaInnotech Corporation). Background staining outside the striatum was subtracted to yield a value for the area of positive staining. Left and right striata were analyzed separately at three similar plane sections along the rostra-caudal axis (Bregma 0.26, 0.74 and 1.18 mm, judged by typical anatomical landmarks) for at least four brains per genotype.

### 2.5 Western blot analysis

SDS polyacrylamide gel electrophoresis (SDS–PAGE), Western blot transfer to Hybond-P filters (GE Healthcare), blocking, incubation with antibodies, washing and detection using enhanced chemiluminescence (ECL or ECL^+^) techniques were carried out as described previously (Buchman et al., 1998; Robertson et al., 2004). Intensities of bands obtained using AlphaImager 2200 were analysed using AlphaEase 2200 Analysis Software (AlphaInnotech Corporation). To extract total striatal proteins, each striatum was homogenized in 150 μl of SDS-PAGE loading buffer followed by heating in the boiling water bath for 5 min. Immunoblotting with mouse monoclonal antibody against β-actin (clone AC-15, Sigma) was carried out using total striatal protein samples diluted to achieve intensity of actin bands within a linear detection range. The original samples were normalized according to results of actin bands densitometry and equal loading was further confirmed by immunoblot analysis of normalized samples with an antibody against another housekeeping protein, either α-tubulin (mouse monoclonal, clone DM 1A, Sigma) or glyceraldehyde 3-phosphate dehydrogenase (GAPDH, mouse monoclonal, clone 6C5, Santa Cruz Biotechnology). Normalized samples were run on 8%, 12% or 16% SDS-PAAG depending on sizes of proteins to be analyzed, transferred to a nylon filter and probed with antibodies against various synaptic markers. Antibodies against α-synuclein (mouse monoclonal, clone 42, BD Transduction Laboratories, diluted 1:500), β-synuclein (mouse monoclonal, clone 8, BD Transduction Laboratories, diluted 1:5000), TH (mouse monoclonal, clone TH-2, Sigma, diluted 1:5000), dopamine transporter (DAT, rabbit polyclonal, Sigma, diluted 1:500), amphiphysin (mouse monoclonal, clone 15, BD Transduction Laboratories, diluted 1:10 000), synaptophysin (mouse monoclonal, clone 2, BD Transduction Laboratories, diluted 1:25 000), synaptotagmin (mouse monoclonal, clone ASV48, QED, diluted 1:5000), SNAP-25 (mouse monoclonal, clone 20, BD Transduction Laboratories, diluted 1:1000), cysteine string proteins (CSP, rabbit polyclonal, Santa Cruz, diluted 1:1000), glial fibrillary acidic protein (GFAP, rabbit polyclonal, Sigma, diluted 1:500), Rab3 (mouse monoclonal, clone 9, BD Transduction Laboratories, diluted 1:2500), rabphilin-3A (mouse monoclonal, clone 47, BD Transduction Laboratories, diluted 1:1000) and sec8 (mouse monoclonal, clone 14, BD Transduction Laboratories, diluted 1:1000) were used. After probing with antibodies against synaptic markers each filter was re-probed with an antibody against one of the housekeeping proteins described above to confirm uniform efficiency of protein transfer. The ratio of intensities of a studied protein band and housekeeping protein band was calculated for each filter and the mean of these ratios for wild type animal samples was considered as 100%. All other ratios were expressed as fractions of this mean allowing to pool together results obtained for each protein from different Western blot filters. Each sample was analyzed for expression of each protein on at least two different Western blot filters with different combinations of other samples.

## 3 Results

### 3.1 Reduced dopamine content in the striatum of aging α-synuclein but not γ-synuclein null mutant mice

**Fig. 1:**
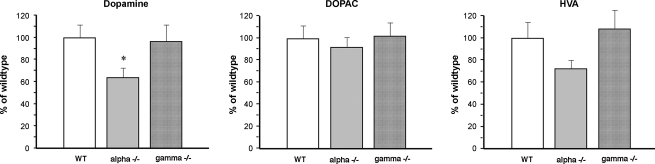
Dopamine and its metabolite levels in striatum of 2-year-old wild type and synuclein null mutant mice. Striatal concentrations (ng/mg protein) of dopamine (DA), 3,4-dihydroxyphenylacetic acid (DOPAC) and homovanillic acid (HVA) in mutant animals were normalized to corresponding mean values for wild type animals (100%) in each experiment. Means ± S.E.M. for 16 wild type (WT), 11 α-synuclein null mutant (alpha−/−) and 10 γ-synuclein null mutant (gamma−/−) animals from two separate experimental cohorts are shown. Statistical analysis revealed significant decrease of striatal DA in α-synuclein null mutant mice (*, *p* < 0.05, one-way ANOVA with post hoc Fisher’s protected *t*-test) but no differences for other neurochemicals and genotypes (*p* > 0.05).

Levels of dopamine and its metabolites in the striatum of aging wild type and null mutant mice were measured using standard HPLC analysis as described in Section 2. Marked reduction of striatal dopamine was detected in 2-year-old α-synuclein null mutant mice (63.8% of the wild type level) but not γ-synuclein null mutant mice (96.4% of the wild type level) when compared to 2-year-old wild type mice (Fig. 1). However, DOPAC levels were the same for all three genotypes and although HVA levels in α-synuclein null mutant mice were lower than in two other genotype groups the difference was not statistically significant (Fig. 1). Consequently, metabolite/dopamine ratios, particularly DOPAC/dopamine, were increased in α-synuclein null mutant mice (Table 1).

**Fig. 2:**
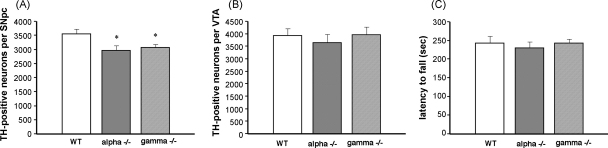
The number of dopaminergic neurons in the substantia nigra and ventral tegmental area of 2-year-old wild type and synuclein null mutant mice and their performance in accelerated rotarod test. Bar chart shows means ± S.E.M. of total number of TH-positive neurons in SNpc (A) and VTA (B). Neurons were counted separately in left and right SNpc and VTA of 9 wild type (WT), 9 α-synuclein null mutant (alpha−/−) and 10 γ-synuclein null mutant (gamma−/−) animals. Statistic analysis revealed significantly reduced number of neurons in SNpc for both types of mutant mice when compared to wild type mice (*, *p* < 0.01, one-way ANOVA with post hoc Fisher’s protected *t*-test) and no difference in number of VTA neurons between all three groups (*p* > 0.05). Bar chat in panel C shows results of an accelerated rotarod test that demonstrated no significant difference (*p* > 0.05) in latency to fall between three groups of 2-year-old mice.

However, the observed reduction of striatal dopamine was not sufficient to impede motor coordination and balance of 2-year-old α-synuclein null mutant mice, as could be judged from their performances in accelerated rotarod (Fig. 2C) that was not different from performances of 2-year-old wild type mice.

### 3.2 No progressive loss of dopaminergic neurons in SNpc of aging α-synuclein or γ-synuclein null mutant mice

In the previous study we have demonstrated that on pure C57Bl6 genetic background both α-synuclein and γ-synuclein null mutant adult mice have small but statistically significant deficit of TH-positive neurons in SNpc comparing to their wild type littermates (Robertson et al., 2004). To assess whether the observed decrease of striatal dopamine in aging α-synuclein null mutant was caused by progressive loss of dopaminergic neurons projecting their axons to the striatum, we counted number of TH-positive neurons in SNpc of 2-year-old wild type, α-synuclein and γ-synuclein null mutant mice. In both groups of null mutant mice these neurons morphologically looked as healthy as in wild type mice (data not shown). Fig. 2A demonstrates that although both α-synuclein and γ-synuclein null mutant mice have less dopaminergic neurons than their wild type littermates at the age of 2 years, these differences (16.5% decrease for α-synuclein and 13.6% decrease for γ-synuclein null mutants) are very similar to those previously reported for 5 day and 6-month-old animals (Robertson et al., 2004). In agreement with previously reported results, we did not find significant difference in number of dopaminergic neurons in VTA of three studied groups of 2-year-old animals (Fig. 2B).

### 3.3 Certain dopaminergic and synaptic marker proteins are downregulated in the striatum of aging α-synuclein null mutant mice

**Fig. 3:**
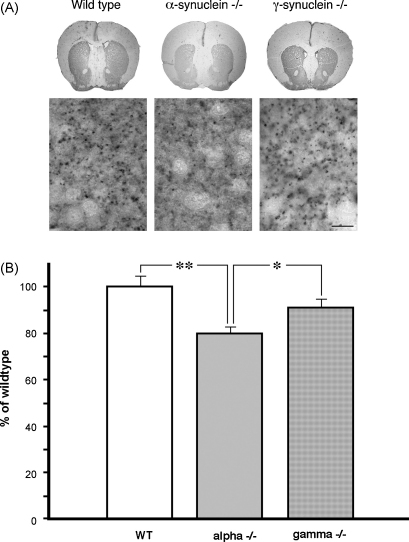
Immunohistochemical detection of tyrosine hydroxylase in the striatum of 2-year-old wild type and synuclein null mutant mice. Representative microphotographs of coronal sections of wild type, α-synuclein and γ-synuclein null mutant mouse brains at the Bregma 1.18 mm level stained with antibody against TH (top panels) and high magnification images that reveal TH-positive fibers in the dorsal striatum (bottom panes, scale bar = 10 μm) are shown (A). Bar chart shows means ± S.E.M. of TH staining densities (B). One-way ANOVA with post hoc Fisher’s protected *t*-test demonstrated that the density is significantly lower in the striatum of α-synuclein (alpha−/−) null mutant mice when compared to wild type (WT) mice (**p < 0.01) or γ-synuclein (gamma−/−) null mutant mice (*p < 0.05).

**Fig. 4:**
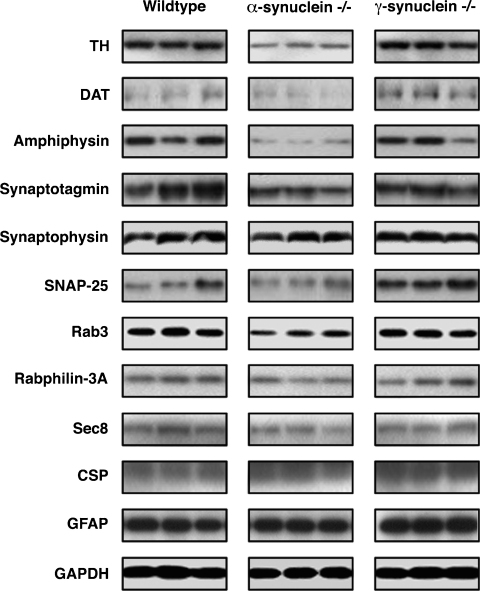
Synaptic marker proteins in the striatum of 2-year-old mice. Representative Western blots of striatal proteins extracted from wild type, α-synuclein and γ-synuclein null mutant mice are shown. Samples extracted from each dissected striatum individually were first normalized using anti-β-actin antibody as described in Section 2. Equal amount of total protein was loaded on each lane and probed with antibody against proteins shown left to each horizontal panel.

Reduced levels of the striatal dopamine in the absence of dopaminergic neuron loss in SNpc might reflect the development of synaptic dysfunction in the nigrostriatal system of aging α-synuclein null mutant mice. To evaluate the dopaminergic innervation of the striatum we stained histological section of this brain region with antibodies against tyrosine hydroxylase. The number of stained fibers was obviously reduced in the dorsal striatum of aging α-synuclein null mutant mice when compared to the striatum of wild type or γ-synuclein null mutant mice of the same age (Fig. 3A). The average density of TH staining was also found to be lower in the dorsal striatum of these mice (Fig. 3B). To confirm these observations we analyzed TH in total protein samples extracted from the striatum of 2-year-old wild type, α-synuclein or γ-synuclein null mutant mice by Western blotting. Consistently with immunohistochemical data, the substantial reduction of TH was observed in the striatum of aging α-synuclein but not γ-synuclein null mutant mice when compared with control wild type mice (Fig. 4 and Table 2). Another marker of dopaminergic terminals, DAT, was also downregulated in aging α-synuclein null mutant mice but upregulated in γ-synuclein null mutant mice. Two synaptic proteins that are not specific to dopaminergic neurons, namely amphiphysin and synaptotagmin, were also found less abundant in the striatum of α-synuclein than in γ-synuclein null mutant or wild type mice, whereas no significant differences were revealed for SNAP-25, Sec8, Rab3, Rabphilin-3A, CSP and synaptophysin (Fig. 4 and Table 2). A slight increase of an astroglial marker GFAP was observed in the striatum of both α-synuclein and γ-synuclein null mutant mice.

### 3.4 Deletion of α-synuclein or γ-synuclein does not affect β-synuclein abundance in the striatum of aging mice

**Fig. 5:**
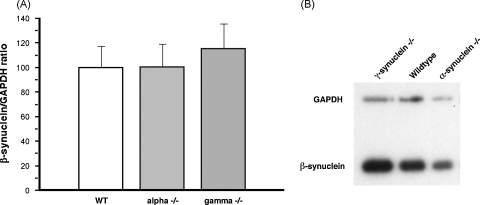
β-synuclein in the striatum of 2-year-old mice. (A) Bar chart shows means ± S.E.M. of β-synuclein/GAPDH band density ratios normalized to the mean ratio for wild type animal samples as 100%. Individual striatum samples from 7 to 9 animals per genotype were analysed by Western blotting. No significant differences were found between groups (*p* > 0.05). (B) A representative Western blot shows analysis of mixtures of the equal amounts of total protein samples from three striata for each genotype. The filters were probed simultaneously with anti-β-synuclein and anti-GAPDH antibody.

It has been previously suggested that other members of the synuclein family might compensate the absence of an inactivated synuclein and compensatory increase of the expression of remaining member(s) of the family in certain brain regions of synuclein null mutant mice has been reported (Chandra et al., 2004; Robertson et al., 2004; Kuhn et al., 2007). To check if observed changes in the striatum of 2-year-old α-synuclein null mutant mice could be linked with age-related reduction of γ-synuclein and, particularly, β-synuclein in the brain of these mice, we assessed striatal levels of these proteins by Western blotting. Although γ-synuclein is not abundant in aging striatum and was barely detectable on Western blots, no changes of this protein level were observed in the striatum of 2-year-old α-synuclein null mutant mice (data not shown). Much more abundant β-synuclein was easily detectable but no difference in the level of this protein in the striatum of 2-year-old wild type, α-synuclein or γ-synuclein null mutant mice was found (Fig. 5).

## 4 Discussion

In human populations the rate of idiopathic Parkinson’s disease as well as many other neurodegenerative conditions strongly correlates with old age. However, molecular mechanisms that make cells in the ageing nervous system, particularly dopaminergic neurons of the nigrostriatal system, progressively more vulnerable to pathological changes are still not well defined. Recent advances in our knowledge about pathogenesis of human neurodegenerative disorders suggest that combinations of various factors are required for the development of pathological changes that lead to manifestation of a disease. α-synuclein dysfunction is one of the best studied factors implicated in the aetiology and pathogenesis of Parkinson’s disease although exact mechanism of this protein involvement in the development and progression of neurodegenerative changes is not clearly understood. As discussed in Section 1, recent evidences suggest that the loss of functional α-synuclein could enhance neuronal and/or synaptic pathology induced by other factors. However, this concept is not directly supported by studies of mice lacking α-synuclein. No sign of neurodegeneration was found in the nervous system of adult α-synuclein null mutant mice (Abeliovich et al., 2000; Specht and Schoepfer, 2001; Cabin et al., 2002; Dauer et al., 2002; Schluter et al., 2003), moreover dopaminergic neurons of these animals were found to be less sensitive to neurotoxic effects of MPTP (Dauer et al., 2002; Schluter et al., 2003; Drolet et al., 2004; Robertson et al., 2004; Fornai et al., 2005), 6-OHDA (Alvarez-Fischer et al., 2008) and paraquat (our unpublished observations). To explain these discrepancies we have suggested that in order to survive without α-synuclein, dopaminergic neurons of substantia nigra pars compacta should keep activity of some pro-survival pathways at elevated level. This makes neurons more robust to certain environmental challenges but only until they are able to sustain required level of compensation. In this study we used old (≥2 years of age) synuclein null mutant mice as a model system to address the question if aging could be a factor that unveil any deteriorating effect(s) of synuclein deficiency on the nigrostriatal system.

In our previous study a small but statistically significant neuronal deficit has been revealed in SNpc of adult α-synuclein and γ-synuclein null mutant mice comparing with wild type mice (Robertson et al., 2004). We found no progressive loss of dopaminergic neurons in SNpc of old animals lacking either of these two synucleins, suggesting that the absence of these proteins does not make dopaminergic neurons more susceptible to death in the aging substantia nigra. However, in the striatum of aging α-synuclein null mutant mice we observed changes that suggested impaired function of dopaminergic neuron synapses. A significant depletion of striatal dopamine in 2-year-old α-synuclein null mutant mice that have not been seen in younger 9-month-old animals (Robertson et al., 2004) indicates age-associated partial loss of striatal dopamine neurotransmission triggered by the absence of α-synuclein but not γ-synuclein. Most probably it is due to reduction of the number of functional dopaminergic synapses, while significantly increased DOPAC/dopamine and slightly increased HVA/dopamine ratio reflect increased dopamine turnover. Similar increase of metabolite/dopamine ratios has been reported in various animal models at the recovery stage that followed lesion of the nigrostriatal system and is believed to reflect a compensatory increase of dopamine turnover in remaining functional synapses (Hefti et al., 1985; Altar et al., 1987; Rose et al., 1989; Pifl and Hornykiewicz, 2006). Reduced density of TH-positive fibers and lower levels of TH and DAT, markers of dopaminergic synapses, that we have observed in the striatum of 2-year-old α-synuclein null mutant compared to wild type or γ-synuclein null mutant mice are consistent with the idea that the loss of α-synuclein function causes partial loss of dopaminergic synapses in the aging striatum. For the majority of studied general presynaptic proteins no significant changes in expression levels were found, suggesting that other types of synapses in the striatum of aging α-synuclein null mutant mice are not affected. The lower level of synaptotagmin is probably not associated with age-related changes because reduction of synaptotagmin mRNA expression has been found previously in various brain regions of young α-synuclein null mutant mice (Kuhn et al., 2007). A reason for the reduction of amphiphysin is not clear and might be associated with downregulation of this protein expression in aging nervous system of α-synuclein null mutant mice.

Results of our previous studies indicated that both α-synuclein and γ-synuclein are required for efficient survival of dopaminergic neurons during critical periods of the substantia nigra development (Robertson et al., 2004). In another study the synaptoprotective role of α-synuclein during early postnatal development has been demonstrated (Chandra et al., 2005) but the involvement of γ-synuclein in this process has not been assessed. Here we demonstrated that α-synuclein but not γ-synuclein is required for effective protection of dopaminergic synapses in aging striatum. This is not surprising because the level of γ-synuclein in the striatum is very low comparing to the level of α-synuclein (Li et al., 2002; Ninkina et al., 2003). Neuroprotective properties of the third member of the family, β-synuclein, have been demonstrated in various experimental systems (Hashimoto et al., 2001, 2004; da Costa et al., 2003; Park and Lansbury, 2003; Fan et al., 2006) and the increased levels of this protein detected in the midbrain of mice lacking α-synuclein or/and γ-synuclein (Robertson et al., 2004) might be linked with neuroprotection. In the striatum, β-synuclein, similarly to α-synuclein, was found predominantly in presynaptic terminals (Shibayama-Imazu et al., 1993; Nakajo et al., 1994; Li et al., 2002; Schluter et al., 2003; Chandra et al., 2004). Therefore, it was feasible to check if the observed changes in the striatum of aging α-synuclein null mutant mice correlated with a reduced level of β-synuclein. However, no difference in the level of this protein in the striatum of all three studied groups of ageing mice was observed, suggesting limited, if any, role of β-synuclein in the development of these changes. Further detailed studies are required to reveal the exact molecular processes, which functional decline in the striatum of aging animals increases the sensitivity of dopaminergic synapses to α-synuclein deficiency.

In conclusion, the results of our studies are in agreement with a proposed synaptoprotective role of endogenous α-synuclein. We have shown that the absence of this protein makes striatal dopaminergic synapses more vulnerable to changes associated with aging of the nervous system. As the first demonstration of an additive effect of aging and α-synuclein depletion on the induction of synaptic dysfunction in the nigrostriatal system this observation casts new light on the molecular and cellular mechanisms involved in pathogenesis of idiopathic Parkinson’s disease.

## Conflicts of interest statement

The authors declare no actual or potential conflicts of interest.

## Figures and Tables

**Table 1:** Metabolite to dopamine concentration ratios in striata of 2-year-old wild type, α-synuclein null mutant and γ-synuclein null mutant mice.

Genotype	DOPAC/dopamine (mean ± S.E.M.)	HVA/dopamine (mean ± S.E.M.)
Wild type	0.20 ± 0.01	0.06 ± 0.01
α-synuclein −/−	0.31 ± 0.04*	0.08 ± 0.01
γ-synuclein −/−	0.22 ± 0.02	0.07 ± 0.01

* *p* < 0.05, one-way ANOVA with post hoc Fisher’s protected *t*-test.

**Table 2:** Levels of protein markers in striata of 2-year-old wild type, α-synuclein null mutant and γ-synuclein null mutant mice expressed as % of wildtype mean.

Marker	Genotype		
	Wild type	α-Synuclein −/−	γ-Synuclein −/−
TH	100 ± 3.77	70.9 ± 8.09^**^	98 ± 9.14
DAT	100 ± 8.74	63.2 ± 7.96*	139.5 ± 11.32*
Amphiphysin	100 ± 6.10	56.3 ± 12.24^**^	94.6 ± 6.91
Synaptotagmin	100 ± 6.24	71.5 ± 9.96^**^	96.5 ± 8.80
Synaptophysin	100 ± 5.28	104.8 ± 6.93	91.2 ± 5.09
SNAP25	100 ± 8.79	97 ± 14.98	115 ± 7.72
Rab3	100 ± 17.23	83.2 ± 13.62	117.5 ± 12.62
Sec8	100 ± 7.20	86.6 ± 5.37	89.6 ± 14.52
CSP	100 ± 2.75	108.9 ± 7.37	109.3 ± 7.43
GFAP	100 ± 3.93	122.0 ± 9.09*	122.7 ± 7.36*

* *p* < 0.05 and ^**^p < 0.01, one-way ANOVA with post hoc Fisher’s protected *t*-test.
